# Perception of Job-Related Risk, Training, and Use of Personal Protective Equipment (PPE) among Latino Immigrant Hog CAFO Workers in Missouri: A Pilot Study

**DOI:** 10.3390/safety2040025

**Published:** 2016-11-09

**Authors:** Athena K. Ramos, Axel Fuentes, Natalia Trinidad

**Affiliations:** 1 Center for Reducing Health Disparities, College of Public Health, University of Nebraska Medical Center, 984340 Nebraska Medical Center, Omaha, NE 68198-4340, USA; 2 Rural Community Workers Alliance, 110 E. 3rd St., Milan, MO 63556-1338, USA

**Keywords:** immigrant farmworkers, animal confinement, swine, health disparities, agricultural health, worker safety, concentrated animal feeding operation (CAFO)

## Abstract

Hog production in the United States is a large industry that has seen dramatic changes over the last few decades. Concentrated animal feeding operations (CAFOs) are growing in number throughout the country. This pilot study explores the perception of risk, receipt of work-related training, provision and usage of personal protective equipment (PPE), and prevention preferences of Latino immigrant hog CAFO workers in Missouri. Forty workers (M age = 36.08 years, SD = 10.04; 92.5% male; 70.0% Mexican) were interviewed. Results indicate that most workers did not perceive their job as dangerous. Limited English proficient workers were significantly less likely to report receiving any work-related training. Although most workers had access to employer provided PPE, usage was inconsistent. As the demographic composition of the farmworker population in the Midwest becomes increasingly comprised of hired immigrant workers, it will be imperative to develop occupational safety and health educational and outreach efforts focused on the needs of these workers.

## 1. Introduction

Hog production in the United States is a $22.5 billion industry [1], and 115 million hogs were produced in the U.S. in 2015 [2]. The hog production industry has changed dramatically over the last few decades, moving from small family farms to large corporate-owned farms [3] that specialize in specific phases of production [4] to reduce production costs and increase economies of scale [5]. These concentrated animal feeding operations (CAFOs) make up a small but increasing number of farms across the U.S. [6]. The U.S. Environmental Protection Agency (EPA) defines an animal feeding operation as a place where “animals have been, are, or will be stabled or confined and fed or maintained for a total of 45 days or more in any 12-month period, and where crops, vegetation, forage growth, or post-harvest residues are not sustained in the normal growing season over any portion of the lot or facility” [7]. Because of the structural shifts toward concentration and phased production, a larger hired workforce is necessary, and many immigrant workers are now filling these jobs at the CAFOs [8–10].

Agriculture is one of the most dangerous industries in the U.S. [6,11] and is considered dirty, dangerous, and demanding (3-D) [12]. All across the country, there is an overrepresentation of immigrants in 3-D agricultural jobs such as hog production [12,13].

Hog production can have serious acute and long-term health effects on farmworkers [8,14,15]. The CAFO environment may intensify health and safety risks due to the increased number of animal units per worker [6]. Because animals are enclosed in a confined area, noise levels can rise above 85 decibels, causing noise-induced hearing loss (NIHL) among workers [16]. Hearing loss can further contribute to workplace accidents and injuries by making it difficult to hear potential warning signs [17,18]. CAFO workers have been found to have chronic or intermittent respiratory problems and nasal symptoms [5,14,19,20]. Workers often report symptoms related with odor such as irritation of the eyes, nose, throat, and headaches [19]. These symptoms may arise due to exposure to hydrogen sulfide, ammonia, volatile organic compounds, particulate matter, and endotoxins commonly found in CAFOs [6,17,20]. These effects may be exacerbated if the worker uses tobacco [6] or other substances. Apart from the health problems that arise due to noise and air emissions, there is an increased risk of zoonotic diseases such as influenza that can be transmitted from hogs to humans [21]. Other effects from working in hog CAFOs include skin irritations, stress, musculoskeletal problems, and nausea [19,20,22].

Often times, immigrant farmworkers are not given any job specific training or safety and health information relating to occupational risks [23]. Workers commonly do not receive any information about personal protective equipment (PPE) that should be worn at the job-site [24]. If workers do receive any information, often it is not in their primary language [23]. Because of language and cultural barriers, workers may not know what risks are inherent in their job and therefore not understand how to protect themselves from job-related risks [9]. Many immigrant farmworkers are socially, economically, and legally vulnerable which may increase their occupational risks [9] and promote under-reporting of workplace hazards and injuries due to fear of losing their job or being undocumented [25].

## 2. Hog Production in Missouri

Missouri is the seventh leading hog producing state in the U.S. [26,27] with an inventory of 3 million hogs [27]. Hog production is responsible for a gross state product of $791 million and 12,663 jobs [2]. To be considered a CAFO, a facility in Missouri must confine more than 1000 animal units, which is equal to 2500 swine [28]. Unfortunately, no data about immigrant hog CAFO workers in the state are available.

## 3. Purpose

The purpose of this analysis is to explore the perception of risk, receipt of work-related training, provision and usage of PPE, and prevention preferences of Latino immigrant hog CAFO workers in Missouri.

## 4. Methods

Data are from a cross-sectional survey conducted with Latino immigrant hog CAFO workers between June and August 2015.

## 5. Study Population

To participate in the study, individuals had to be at least 18 years old, be an immigrant of Hispanic/Latino descent, and currently work in a hog CAFO in Missouri. Workers were recruited to participate in this study through convenience sampling methods. In Audrain county, workers were recruited through door-to-door outreach in immigrant neighborhoods that were identified by community leaders as being places where most CAFO workers lived. In Linn and Sullivan counties, workers were recruited through their participation in safety and workers’ rights workshops and English as a second language (ESL) classes, which could have impacted workers’ experiences and responses. A total of forty Latino immigrant hog CAFO workers from Audrain, Linn, and Sullivan counties in Missouri participated in the study.

## 6. Procedures

The University of Nebraska Medical Center (UNMC) Center for Reducing Health Disparities partnered with the Rural Community Workers Alliance (RCWA), a worker advocacy non-profit organization based in Milan, Missouri, through a community-based participatory research process to design and facilitate the implementation of the study. Data were collected by two bilingual and bicultural research team members (one academic partner and one community partner) at participants’ homes. The study methodology and the rights of research participants were explained to each participant and informed consent was obtained. Verbal face-to-face interviews with participants were conducted to administer the Immigrant CAFO Worker Survey. All study materials were available in English and Spanish, and participants could choose to participate in the language in which they were most comfortable; however, only one participant chose to respond in English. Participants were given a $10 gift card for their participation in the study, which was approved by the UNMC Institutional Review Board.

## 7. Measures

The Immigrant CAFO Worker Survey consisted of 130 questions divided into six sections: (1) health status; (2) occupational health and perception of risk; (3) emotional health; (4) stress; (5) prevention; and (6) demographics. Survey content included reliable and valid standardized measures as available. The cultural and linguistic appropriateness of the survey instrument was reviewed by a representative of the RCWA who has over ten years of experience working with rural Latino agricultural communities in the U.S. and has an agricultural education background from Guatemala.

### 7.1. Perception of Occupational Risk

Perception of occupational risk was measured with one question, “How dangerous do you feel your job is?”. Response options were categorical and included: not at all dangerous (0); a little bit dangerous (1); dangerous (2); and very dangerous (3). Response categories were dichotomized for part of this analysis—not at all dangerous and a little bit dangerous were collapsed into “not dangerous” and dangerous and very dangerous were collapsed into “dangerous”.

### 7.2. Training

Job-related training was measured through the question, “Have you ever received any health and/or safety training from your current employer?”. Response options were dichotomous: no (0) and yes (1). If a participant responded that they had received training, a series of follow-up questions were asked including how often training was provided and in what language was it provided.

### 7.3. Personal Protective Equipment Provision and Usage

PPE provision was assessed through the question, “Does your employer provide any type of personal protective equipment (PPE) to you for your job?”. Response options were dichotomous: no (0) and yes (1). If the participant responded affirmatively that their employer did provide PPE, then they were asked which type of PPE was provided such as respirator, ear plugs/hearing protection, uniforms/coveralls, boots or shoe covers, gloves, goggles, or head or hair covers. PPE usage was measured by the question, “When you are exposed to hazards on the job, how often do you wear the following: face mask/respirator, ear plugs/hearing protection, uniforms/coveralls, boots or shoe covers, gloves, safety goggles, and head or hair covers?”. Response options were based on standard conventions [29] and included never (0); some of the time (1); and all of the time (2).

### 7.4. Prevention Preferences

Participants were asked a series of questions regarding health and safety promotion and prevention opportunities. Some of the questions included: “Is health and safety important to you?” and “Would you like to receive more information on health and safety related to your job?”. Response options were dichotomous: no (0) and yes (1). Additionally, participants were asked, “How would you prefer to receive this information?” and “In which language do you prefer to receive health and safety information related to your job?”. Response options for the first question included training at the workplace (0); training in the community (1); or online/internet (2); and response options for the second question were English (0); Spanish (1); either language (2); or other (3) with the option to specify the language which they would prefer.

### 7.5. Demographic Variables

Workers’ age and number of hours worked per week were continuous variables. English language proficiency was measured by a single question, “How well do you speak English?”. There were four original response options which were later dichotomized into well or very well (0) and not well or not at all (1) to create a variable representing limited English proficiency.

## 8. Analytic Approach

The Statistical Package for the Social Sciences (SPSS) version 23.0 was used to analyze the data. Descriptive statistics including frequencies for categorical variables as well as means and standard deviations for continuous variables were calculated. Bivariate analyses were conducted. Chi square tests, particularly Fisher’s Exact test (two-sided), were used to measure associations between categorical variables due to the small sample size.

## 9. Results

The final sample was: 92.5% male, 69% under age 40 (M = 36.08, SD = 10.04), and 77.5% with less than a high school education (Table 1). Most participants were from Mexico, but some workers were also from El Salvador and Guatemala. Half of participants had no prior experience working with hogs either in the U.S. or in their country of origin, 85% had been employed in the industry less than three years, and 77.5% worked with sows or piglets. On average, participants worked more than 50 hours per week (M = 52.72, SD = 10.38).

Most workers did not perceive their job to be dangerous with 67.5% responding that their job was not at all dangerous or just a little bit dangerous. Fisher’s Exact test was performed and a significant relationship was found between reporting an occupational injury and perception of risk, *p* = .01 (Table 2). Workers who reported that they had been injured were more likely to also report that their job was dangerous or very dangerous.

Nearly two thirds of workers reported that they received some form of job-related training from their employer. Of those who did report receiving training, 25% of participants stated that it was only provided in English. Fisher’s Exact test was performed and a significant relationship was found between workers reporting receipt of training and English language proficiency, *p* = .02 (Table 2). All of the workers who spoke English well or very well reported receiving training, but only about half of the workers (53.1%) who had limited English proficiency reported receiving any job-related training.

Although most workers had access to employer provided PPE, usage was inconsistent. Sow barn and nursery pig workers reported never using respirators, hearing protection, and safety goggles more often than those employed in finishing barns, building maintenance, or washing tasks (Table 3).

Amongst participants, 28.2% believed that they had health problems as a result of working with hogs. Approximately one third (32.5%) of workers had been injured on the job. The most frequently cited physical injuries were related with the leg, knee, or hip (30.8%), hand or wrist (23.1%), and eyes (15.4%).

Nearly all participants agreed that health and safety was important to them, and 82.1% would like more occupational health and safety information. Providing this information in person either at the jobsite or in the community was preferred, rather than online. Eighty-seven percent of workers preferred to receive this information in Spanish, and 7.5% noted that it would be helpful to have information in Mayan languages such as K'iché or Q’anjob’al.

## 10. Discussion

Two-thirds of participants did not think their job was dangerous, and having a work-related injury increased a worker’s perception of risk, as an injury is a clear reminder of personal susceptibility. Other reasons, such as cultural factors, may influence whether or not a worker reports their job to be dangerous. Arcury et al. (2006) noted that Latino farmworkers may interpret the meaning of personal susceptibility differently [22]. Cultural expectations of being tough and strong may negatively impact perception of risk and implementation of appropriate preventative behaviors such as the use of PPE among Latino immigrant workers. Underlying cultural issues and the role they play in the decision to use safety equipment need to be further explored within this worker population.

All workers, including Latino immigrant CAFO workers, have a right to occupational health and safety information. According to the International Labour Organization’s Safety and Health in Agriculture Convention (C-184), Article 8, workers have a right “to be informed and consulted on safety and health matters” [29]. However, more than one third of workers in our sample reported not receiving any job-related training from their current employers. Orientation and training are imperative, especially when workers are new to the industry, such as the 50% of respondents from this sample who reported no previous experience working with hogs. The agricultural industry, safety organizations, and workers’ centers should use culturally and linguistically appropriate training materials to eliminate information barriers for immigrant farmworkers. For example, one starting point for training new workers could be the Pork Quality Assurance Plus (PQA Plus) program that was recently released by the National Pork Board, given that it is readily available in English and Spanish [30]. Any training programs should be supplemented with consistent health and safety messaging on the job, such as weekly safety briefings or a short daily (tailgate) safety discussions right before workers start their shift [31].

Workers had a high rate of consistent use of PPE items such as coveralls, boots, and gloves, which may be due to biosecurity concerns. Although additional PPE was provided by employers, workers did not use those other items such as respirators, hearing protection, and goggles consistently when exposed to hazards. Workers may understand immediate dangers or nuisances present in the work environment, such as a chemical exposure to the eyes during washing; hence, washing and maintenance workers were most likely to report using safety goggles all of the time. Although workers may understand immediate dangers or nuisances, they may downplay their susceptibility to long-term occupational health issues such as NIHL or respiratory conditions, which may partly explain some of the patterns of respirator and hearing protection use in our study. These findings are consistent with previous research which has demonstrated that Latino workers often focus on the acute symptoms related to occupational exposures [22,32], rather than both the immediate and long-term effects.

Having PPE available and encouraging its use is important [22], however, conducting regular safety audits to check to ensure that workers are using the PPE and donning it properly are necessary. Anecdotally, workers mentioned during the interviews that they saw the PPE at the workplace, but did not know what it was for or how to use it. Because workers’ safety and health are of utmost concern, both workers and supervisors could be evaluated for their consistent use of PPE and demonstrating safety skills. In the future, our team will be working to adapt materials to educate workers about appropriate PPE and how to properly use it. The proper use of PPE among farmworkers could reduce the risk of illness and injury and promote a healthy work environment.

This pilot study highlights an example of how valuable a collaboration between academia and a community-based organization can be for assessing agricultural safety and health among vulnerable populations. Without the partnership, recruitment for this study would have been more difficult. RCWA brought community-based knowledge and trust inherent in established relationships with vulnerable immigrant worker communities. Creating, maintaining, and expanding campus-community collaborations may lead to mutually beneficial outcomes like stronger preventative outreach and education, enhanced response to emerging threats, more relevant research, and effective interventions.

## 11. Limitations

This was a pilot study, and therefore, it is limited by a small sample size. It focused specifically on Latino immigrant workers and cannot make any conclusions about other groups of immigrant farmworkers who may not be Latino. The study also specifically focused on hog production workers. Conditions may be different in other types of CAFOs, such as in poultry production or cattle feedlots. The study lacked a comparison group, and there is the possibility of selection bias given the non-probability sample and different types of recruitment methods employed in the three counties. Because the data were drawn from individuals, there is always the risk of social desirability bias in responses and perhaps an over-reporting of positive safety practices. PPE use of all workers was assessed using a standard list, rather than a specialized listing based on specific job responsibilities. Finally, information was sought only from CAFO workers, not from employers; therefore, workers’ reports cannot be corroborated.

## 12. Conclusions

The National Institute of Occupational Safety and Health (NIOSH) has identified immigrant farmworkers as a vulnerable population [33]. As the demographic composition of the farmworker population in the Midwest becomes increasingly comprised of hired immigrant workers, it will be imperative to develop occupational safety and health educational and outreach efforts focused on the needs of these workers in order to promote the health and well-being of the agricultural labor force. More research is needed to better understand both the occupational safety and health assets and challenges that are faced by these workers, including the impact of culture on how safety and health risks are perceived among immigrant farmworkers in the Midwest. We plan to continue this line of research using community-engaged, mixed methods designs to explore opportunities for occupational health promotion among Latino immigrant CAFO workers.

## Figures and Tables

**Table 1:** Demographic characteristics of participants.

Variable	N (%)
Sex	

Male	37 (92.5)
Female	3 (7.5)

County of Residence	

Audrain	34 (85.0)
Linn	1 (2.5)
Sullivan	5 (12.5)

Education	

Less than High School	31 (77.5)
High School Graduate	8 (20.0)
Some College or Technical Training	1 (2.5)

Income	

Less than $10,000	4 (10.8)
$10,000–$25,000	19 (51.4)
$25,001–$50,000	11 (29.7)
More than $50,000	3 (8.1)

Primary Language Spoken in the Home	

Mainly Spanish	28 (70.0)
English and Spanish Equally	5 (12.5)
Mainly English	1 (2.5)
Indigenous Languages	6 (15.0)

English Proficiency	

Not at All	9 (22.5)
Not Well	23 (57.5)
Well/Very Well	8 (20.0)

Previous Experience Working with Hogs	

Yes	20 (50.0)
No	20 (50.0)

Length of Employment at Current CAFO	

Less than 1 Year	17 (42.5)
1–3 Years	17 (42.5)
More than 3 Years	6 (15.0)

Type of Work at Current CAFO	

Sow Barn	23 (57.5)
Nursery Pigs	7 (17.5)
Finishing	2 (5.0)
Maintenance/Washing/Other	8 (20.0)

**Table 2:** Chi square tests of associations between study variables.

Risk Perception			
Job-Related Injury	Dangerous N (%)	Not Dangerous N (%)	*p* Value
Injured	8 (61.5)	5 (38.5)	.01
Not Injured	5 (18.5)	22 (81.5)	

**Reported Receipt of Training**			

**Language**	**Received Training N (%)**	**Did Not Receive Training N (%)**	***p* Value**

English Proficient	8 (100.0)	0 (0.0)	.02
Limited English Proficient	17 (53.1)	15 (46.9)	

**Table 3:** Provision and frequency of use of personal protective equipment (PPE).

Type of PPE	PPE Provided By Employer (All Workers) N (%)	Sow Barns (*n* = 23)			Nursery Piglets (*n* = 7)			Finishing (*n* = 2)			Washing/Maintenance (*n* = 8)		

		Never N (%)	Sometimes N (%)	All of the Time N (%)	Never N (%)	Sometimes N (%)	All of the Time N (%)	Never N (%)	Sometimes N (%)	All of the Time N (%)	Never N (%)	Sometimes N (%)	All of the Time N (%)
Respirator	37 (92.5)	2 (8.7)	12 (52.2)	7 (30.4)	1 (14.2)	3 (42.9)	3 (42.9)	0 (0.0)	2 (100.0)	0 (0.0)	0 (0.0)	5 (62.5)	2 (25.0)
Hearing Protection	38 (95.0)	3 (13.0)	11 (47.8)	8 (34.8)	2 (28.6)	2 (28.6)	3 (42.9)	0 (0.0)	2 (100.0)	0 (0.0)	0 (0.0)	2 (25.0)	5 (62.5)
Uniforms/Coveralls	38 (95.0)	0 (0.0)	2 (8.7)	21 (91.3)	0 (0.0)	0 (0.0)	7 (100.0)	0 (0.0)	0 (0.0)	2 (100.0)	0 (0.0)	1 (12.5)	6 (75.0)

Rubber Boots/Disposable Shoe Covers	39 (97.5)	0 (0.0)	2 (8.7)	21 (91.3)	0 (0.0)	0 (0.0)	7 (100.0)	0 (0.0)	0 (0.0)	2 (100.0)	0 (0.0)	0 (0.0)	7 (87.5)

Gloves	39 (97.5)	0 (0.0)	3 (13.0)	20 (87.0)	0 (0.0)	0 (0.0)	7 (100.0)	0 (0.0)	1 (50.0)	1 (50.0)	0 (0.0)	1 (12.5)	6 (75.0)
Goggles	36 (90.0)	3 (13.0)	13 (56.5)	6 (26.1)	1 (14.2)	4 (57.1)	2 (28.6)	0 (0.0)	2 (100.0)	0 (0.0)	0 (0.0)	3 (37.5)	4 (50.0)
Hair Covers	13 (32.5)	17 (74.0)	2 (8.7)	1 (4.3)	4 (57.1)	3 (42.9)	0 (0.0)	2 (28.6)	0 (0.0)	0 (0.0)	4 (50.0)	2 (25.0)	1 (12.5)
